# A Progressive Search Method for Roundness Evaluation Based on Minimum Zone Criterion

**DOI:** 10.3390/mi16040467

**Published:** 2025-04-15

**Authors:** Jian Mei, Binbin Li, Guohua Hu, Chuanzhi Fang, Sheng Zhang, Juan Zheng, Qian Zhang, Lei Hong, Qiangxian Huang

**Affiliations:** 1 School of Advanced Manufacturing Engineering, Hefei University, Hefei 230601, China; meijian@hfuu.edu.cn (J.M.); li787397967@163.com (B.L.); zhangsheng@hfuu.edu.cn (S.Z.); zhj013062@hfuu.edu.cn (J.Z.); zqhd@hfuu.edu.cn (Q.Z.); hongleio@hfuu.edu.cn (L.H.); 2 School of Mechanical Engineering, Hefei University of Technology, Hefei 230009, China; 3 School of Mechanical Engineering, Anhui Institute of Information Technology, Wuhu 241000, China; fangchuanz@163.com; 4 School of Instrument Science and Opto-Electronic Engineering, Hefei University of Technology, Hefei 230009, China; huangqxhfut@163.com

**Keywords:** roundness error evaluation, progressive search method, minimum zone circle, search circle model

## Abstract

With the rapid development of micro-machining technology, the feature size of object parts becomes smaller whilst the roundness tolerance must be critically verified at sub-micrometers or nanometers. Therefore, establishing a method is critically important for evaluating roundness errors to guarantee the machining quality. A progressive search method is proposed based on the minimum zone criterion in the Cartesian coordinate system in this paper. The least-square center of the measured points is used as the initial reference center by establishing a search circle model for progressively approaching the minimum zone circle center. To testify to the feasibility and performance of the proposed method, comparison and simulation experiments are implemented. The results demonstrated that the progressive search method is effective, reliable, and can evaluate roundness error accurately and quickly with not more than 0.1 s and 10 times.

## 1. Introduction

In the field of precision devices, precision machinery, and high-tech equipment, the form error of high-precision round components is an important factor that determines the machining quality. Roundness is an elemental and essential geometrical feature of mechanical parts, which directly affects the assembly and functionality of the parts, such as cylindrical components [1]. The machining precision and roundness measuring techniques have been increasingly required in recent years [2,3]. As a result, roundness error must be guaranteed accurately. Thus, a simple, accurate and effective method for evaluating the roundness error plays a more and more important role. According to ASME and ISO standards [4,5], the roundness error must meet the minimum zone criterion. The entire profile should be contained by two concentric circles, whilst the radius difference should be minimal.

The four primary techniques employed for roundness evaluation are least square circle (LSC), minimum circumscribed circle (MCC), maximum inscribed circle (MIC), and minimum zone circle (MZC). The LSC is based on mathematical analysis that can minimize the sum of squared residuals of the radial deviations between the measured points and the fitted circle [6,7]. The LSC was verified to be efficient in some areas, such as software analysis and mathematical models, so it is widely used as the approximate result. Nevertheless, the LSC is not satisfied with the minimum conditions of form error evaluation because it introduces uncertainty and error in the evaluation result. So the roundness error value obtained is not the minimum. The MCC is defined as a circle with a minimal radius that encloses all the measured points, where at least one point lies on its circumference. In contrast to MCC, the MIC is defined as the circle with the maximal radius that can be contained within all the measured points, where at least one point lies on its circumference [8]. The roundness errors of the MCC and MIC obtained are not the minimum as well because only one geometric boundary is constrained. According to the presentation of ISO standards [5], the MZC is the only one that can meet the requirement of the minimum conditions criterion. The core technology of MZC is to search two concentric circles with the minimum radius deviation which can involve all the measured points of the circle surface.

Some studies on the evaluation of the roundness error have been proposed in recent years. Chen [9] proposed a detection system for measuring roundness and an optimization algorithm to solve the reference circle of MZC. A computational software was developed based on the basic mathematics of circles to evaluate the roundness error using the MZC method [10]. In the past decade, Voronoi diagrams have been constructed to solve the minimum zone solutions with the help of the rapid development of computational geometric techniques. Based on the Voronoi diagrams, Huang [11] proposed a mathematical model to determine the critical measured points that give the exact minimax solutions for the roundness evaluation. Samuel [12] applied computational geometric techniques with convex hulls of Voronoi diagrams for solving the solutions of roundness evaluation. A MZC algorithm was proposed by employing a computational geometry approach through the Voronoi diagrams to evaluate roundness [13]. Some other computational geometry-based algorithms have also been developed for roundness evaluation [14,15,16,17]. A computational geometry algorithm that can reduce candidate points for establishing minimum zone limacons was proposed in roundness evaluation [14]. The curvature technique was employed to reduce the computation to solve the roundness problems by Li and Shi [15]. Feng [16] proposed a combinatorial search algorithm based on the unique integrated property point subset for determining the roundness. Goch [17] applied Tschebyscheff approximation algorithms to determine correctly standardized form tolerances of circle objects. It was verified that the algorithms can be applied to circles, cylinders, and other geometry elements. Zhu [18] proposed the steepest descent algorithm for circularity evaluation. By calculating the minimum translational distance between two convex polygons, the descent direction can be determined for the optimal solution. Lei [19] proposed a geometry optimization algorithm of the MZC method to search the object point with the help of a reference regular square. A model of intersecting chords was proposed to solve the roundness error by Liu [20]. A particle swarm optimization (PSO) algorithm was proposed to evaluate roundness by decreasing the inertia weight linearly during the iterative process based on MZC [21]. Sun [22] applied PSO-based methods to compute MIC, MCC, and MZC problems for determining roundness errors under a machine vision system. The impact of the inertia weight, maximum velocity, and the number of particles on the performance of the particle swarm optimizer was analyzed. A chord-angle method is proposed by determining the chord and its two corresponding minimum angles to solve the roundness error evaluation by Li [23]. Some ideas of roundness profile strategy are used by measuring equipment such as cylindricity instruments and coordinate measuring machine (CMM) [24]. Jiang [25] proposed a novel algorithm that can reduce the uncertainty of fitting the sampled points to solve the solutions of roundness zones. The algorithm has been verified to be robust and reliable for roundness evaluation with a relatively small number of data points. Some other optimization methods have also been introduced based on genetic algorithms (GAs) [26,27,28,29]. Lai [26] proposed a heuristic approach based on GAs that can apply the numerical-oriented genetic operator to model form errors for roundness evaluation. Wen [27] proposed a canonical GA for the circularity error unified evaluation, which does not require genetic parameters. Therefore, it is very convenient to use in engineering metrology. Rossi proposed a combination of five genetic parameters [28] and optimal sampling strategy [29] methods for minimum zone roundness evaluation. They can reduce the computation time and provide sufficient accuracy. The approaches demonstrated above are mainly aimed at optimizing the minimum zone circle instead of determining an accurate search with a few iterations and little time for the MZC center. In this paper, a progressive search method is proposed that can determine the roundness error accurately and effectively with a few iterations and little time.

The proposed method is fully in accordance with the criteria of minimum conditions. The search process mainly consists of two parts: Firstly, a quasi-MZC center (very close to the MZC center) is obtained by establishing the search circle model. Secondly, the MZC center is determined by employing the quasi-center as the reference center to obtain the two concentric circles.

## 2. Control Model of Determining MZC with Measured Points

**Figure 1:**
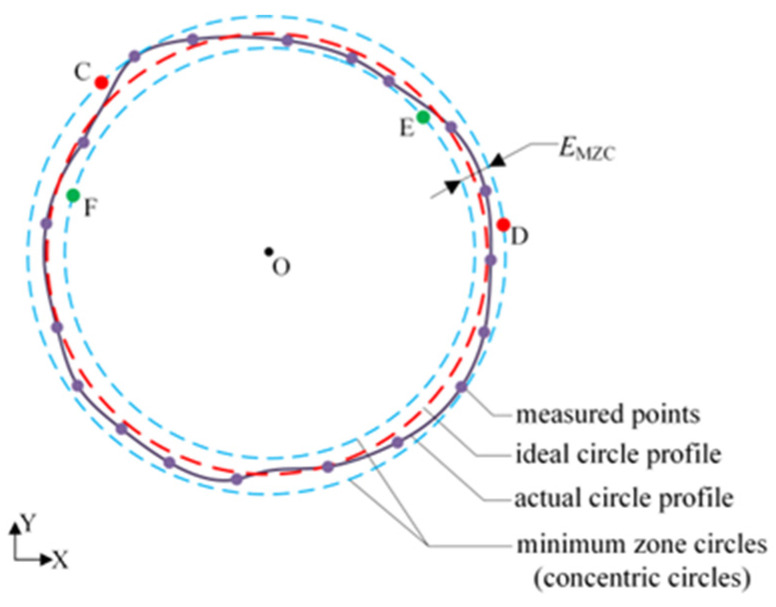
Geometric structure of the MZC roundness evaluation and the 2+2 model. O is the center of the minimum zone circles. C and D are the control points on the outer circle and E and F are the control points on the inner circle.

The geometry of the MZC roundness error evaluation is schematically demonstrated in Figure 1, whose roundness error is denoted as *E_MZC_*. Then, the main job of the roundness error evaluation is to obtain the minimum zone circle accurately, namely two concentric circles with a minimum radius deviation. Generally, the minimum zone circle can be described uniquely by four control points on two circles. Consequently, it is necessary to determine four parameters associated with the radii of their inner and outer circles, as well as the common center (referred to as the MZC center), within the Cartesian coordinate system.

Four equations can be established by utilizing these four points. The distribution of control points can be described as an M+N model, which means that the number of points on the outer circle is M and the number of points on the inner circle is N [11]. There are three combinations of the M+N model, i.e., 3+1, 1+3, and 2+2 models. As the overview in Part 1 and in accordance with the criteria determining the concentric circles, only the 2+2 model can be satisfied with MZC conditions [10,30], as shown in Figure 1.

## 3. Basic Theory of the Progressive Search Method

**Figure 2:**
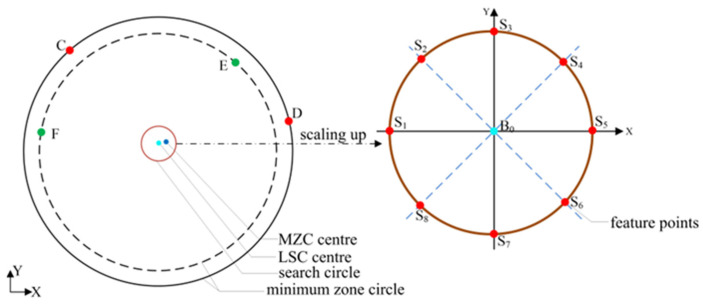
The structure of the feature points on the search circle model.

As the presentation in Section 2, searching for the four control points of two minimum concentric circles can determine the MZC center. Then, the roundness error can be obtained. It is easily operated by searching all the combinations of four points. However, the difficulty will be increased exponentially with hundreds or thousands of measured points. To solve this problem, a progressive search method is proposed to enhance search efficiency and guarantee search accuracy. It is well understood that the object center of MZC is close to the LSC center. Assuming a few reference centers are located between the LSC center and the MZC center, the smaller the roundness error is, the closer the corresponding center is to the MZC center. Therefore, the search direction can be determined accordingly. The entire two-dimensional space must be explored to prevent convergence on a locally optimal solution. Given this, a search circle model, which is constructed based on the LSC center with a radius equal to the LSC roundness error (*E*_0_), is introduced to achieve this goal. It can be easily deduced that the MZC center is located inside the circle, as shown in Figure 2. Certain feature points on the circle are identified to establish the search direction for progressively approaching the MZC center.

By utilizing the x and y coordinate axis in conjunction with the quadrant bisector line based on B_0_, a total of eight intersection points are simultaneously generated within the search circle, which are subsequently identified as feature points. Hypothetically, the eight feature points are, respectively, regarded as the imaginary center of the measured circle, then eight roundness errors can be calculated, i.e., radial extreme differences.

**Figure 3:**
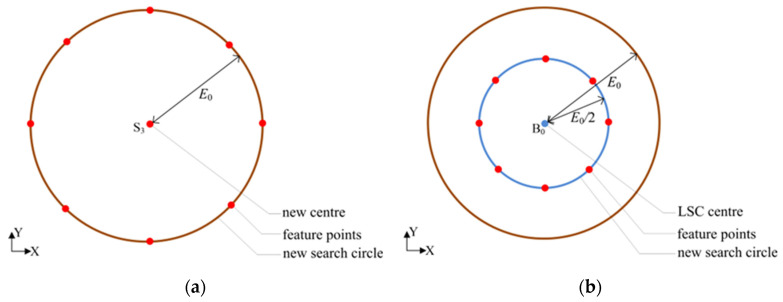
(**a**) A new search circle with a previous feature point as its center; (**b**) A new search circle with a center unchanged and half of the radius.

The LSC center is employed as the initial reference center and the LSC roundness error is regarded as the initial reference error (*E*_0_). The search direction is determined by comparing the eight roundness errors with the reference roundness error. If the minimum value of the eight roundness errors is less than *E*_0_, then the corresponding feature point (e.g., S_3_) is set as the reference center of the new search circle, whose radius remains *E*_0_, as shown in Figure 3a. Then, the reference center is replaced by point S_3_. Similarly, eight new feature points are redefined and their corresponding roundness errors can be obtained. Then, the comparative process is repeated.

On the contrary, if the minimum value of the eight roundness errors is not less than *E*_0_, it indicates that the search circle radius is excessively large. Then, the bisection method is applied by setting the radius of the new search circle to be *E*_0_/2 whilst the center remains unchanged, as shown in Figure 3b. Similarly, eight new feature points and their corresponding roundness errors can be obtained. Then, the comparative process is repeated.

**Figure 4:**
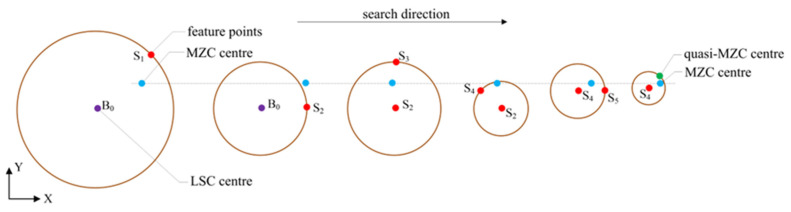
The process of searching quasi-MZC center.

The search execution procedure mentioned above is depicted schematically in Figure 4. Considering the right search direction and fast convergence, if the result is not less than the one of the last step, then the search execution is terminated. After the algorithm converges to a stable state, the point with minimum roundness error is used as a quasi-MZC center.

To obtain an accurate MZC center, the four control points should be identified. Considering that the quasi-MZC center almost coincides with the true circle center, it is used as the approximate center to search for the control points in the algorithm. After the control points are determined, the accurate center can be calculated. Assuming that the coordinates of the four control points are set to be C(*x_C_*, *y_C_*), D(*x_D_*, *y_D_*), E(*x_E_*, *y_E_*), and F(*x_F_*, *y_F_*), then the center coordinates (*x_M_*, *y_M_*) of the concentric circles based on the 2+2 model and outer–inner–outer–inner principle [27] can be obtained by the Equation (1) as follows.

(1) $$\left[\begin{array}{c} x_{M}\\ y_{M} \end{array}\right]={\left[\begin{array}{cc} x_{D}-x_{C} & y_{D}-y_{C}\\ x_{F}-x_{E} & y_{F}-y_{E} \end{array}\right]}^{-1}\cdot \left[\begin{array}{c} 0.5(x_{D}^{2}-x_{C}^{2}+y_{D}^{2}-y_{C}^{2})\\ 0.5(x_{F}^{2}-x_{E}^{2}+y_{F}^{2}-y_{E}^{2}) \end{array}\right]$$

## 4. Implementation

According to the distribution of feature points based on the MZC model, there must be two points located on the outer circle and two points on the inner circle, respectively. Surely, the farthest point and nearest point from the object center are both involved among the four points. So the farthest and nearest point can be determined firstly, whilst the difference in the distance between the two points and the object center is ensured to be minimal.

The program was mainly designed to search the MZC center by using MATLAB R2014a software. The general framework for the computation program, which mainly comprises five parts, namely the calculation of the LSC center and roundness error, the construction of the search circle model, the determination of the quasi-MZC center, the determination of the control points, and the MZC center and the calculation of the MZC roundness error.

### 4.1. Calculating the Coordinate of the LSC Center and Roundness Error

The primary step is to determine the two important values of the LSC center and roundness error for constructing a search circle. The fitting method of the least square circle is a mature mathematical analysis technique. The object function is optimized by minimizing the sum of the square of the error, which can be denoted as follows:

(2) $$f(x,y)=min\sum_{i=1}^{N}{\left(\sqrt{{\left(x_{i}-x\right)}^{2}-{\left(y_{i}-y\right)}^{2}}-R\right)}^{2}$$

where (*x*, *y*) and (*x_i_*, *y_i_*) are the coordinates of the LSC center and the measured point, *N* is the number of measured points, and *R* is the radius of the LSC. After calculation, the LSC center B_0_ and roundness error *E*_0_ are obtained.

### 4.2. Constructing a Search Circle Model

The search circle is constructed by employing the LSC center and roundness error as the initial center and radius. The initial center and radius are denoted as B_0_ and *E*_0_, respectively. The detailed review has been mentioned in Section 3.

### 4.3. Obtaining the Quasi-MZC Center

The measured points are denoted as P*_i_* (*i* = 1, 2, …, *N*) and the eight feature points are denoted as S*_j_* (*j* = 1, 2, …, 8). The coordinates of the measured points are denoted as (*x_i_*, *y_i_*), whereas those of the feature points are denoted as (*x_j_*, *y_j_*). Then, the distance between all the measured points and each feature point can be calculated according to Equation (3). Therefore, the maximum and minimum radius and the extreme difference between all the measured points and each feature point can be obtained. Then, the minimum extreme difference, denoted by *E_min_*, is derived from the eight extreme differences. The feature point that corresponds to *E_min_* is denoted as O_M_ (*x_M_*, *y_M_*).

(3) $$D_{PiSj}=\sqrt{{\left(x_{i}-x_{j}\right)}^{2}-{\left(y_{i}-y_{j}\right)}^{2}}$$

If *E_min_* ≤ *E*_0_, then a new search circle, which has a center that is replaced by O_M_ with an unchanged radius, is established. If *E_m_* > *E*_0_, then the radius decreases to *E*_0_/2, and the center remains unchanged. The termination condition of the searching process is set to *E_min_* ≥ *E_m_*, where *E_m_* is the *E_min_* of the last step. Consequently, the quasi-MZC center is obtained and denoted by O_1_.

### 4.4. Determining the Control Points of the Minimum Zone Circles

**Figure 5:**
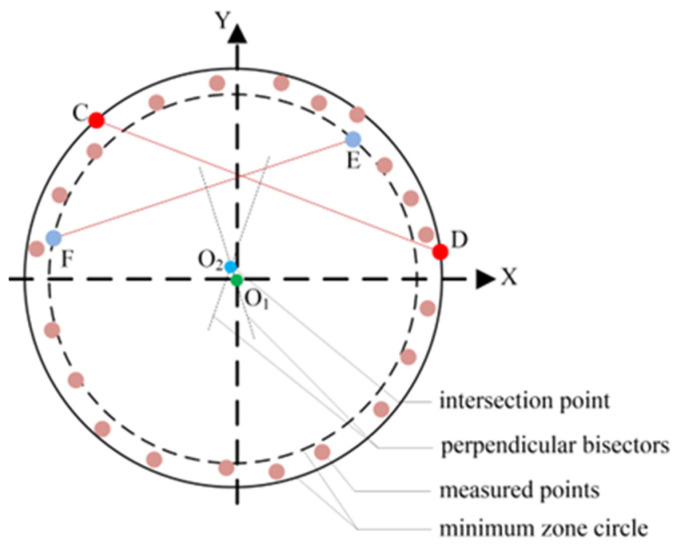
The strategy to determine the stable crossing structure formed by outer chord CD and inner chord EF.

To determine the MZC center, the center O_1_ is used as the initial reference center. With all the measured points, the two farthest and nearest points from the initial center can be calculated by Equation (2). According to the outer–inner–outer–inner principle and Equation (2), the sub-nearest point is also obtained. Two chords on each circle are constructed and a crossing structure is formed by the four points, as shown in Figure 5. Using Equation (1), the intersection point O_2_ of two perpendicular bisectors from two chords can be calculated. Then, determine whether the remaining measured points are contained within the two concentric circles. Firstly, if there were measured points inside the inner circle, the sub-nearest point from the center O_2_ is employed to replace the sub-nearest point of the last step. Secondly, if there were measured points outside the outer circle, the sub-farthest point from the center O_2_ is used to replace the sub-farthest point of the last step. The replacement strategy is completed by measured points located neither inside the inner circle nor outside the outer circle.

**Figure 6:**
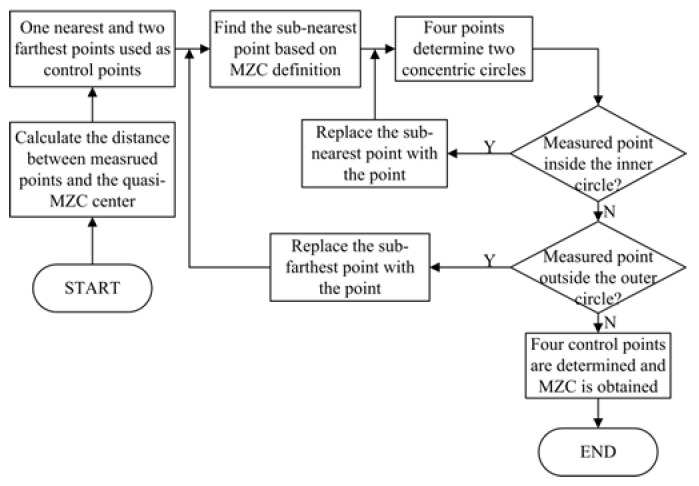
The flow chart to determine the MZC center based on the 2+2 model.

Repeat the search process above until the crossing structure reaches a stable state, i.e., the four points remain unchanged [31]. Then, the four points regarded as the control points are determined. The flow chart of the search process is depicted in Figure 6.

### 4.5. Calculating the MZC Roundness Error

Through Section 4.4, four control points of MZC form two stable chords consequently. Then, the intersection point of perpendicular bisectors of stable crossing structure is the MZC center. The value of the MZC center and roundness error can be calculated as a result.

## 5. Performance Verification of Progressive Search Method

### 5.1. Simulation Experiment Verification by Building Data

A specified circle, whose radius is 1 mm and the roundness error is 10 μm, is artificially established by considering the randomness error (noise) in the Cartesian coordinate system to testify to the performance of the proposed method. The radii of the outer circle and the inner circle based on MZC are determined to be 1.01 mm and 1 mm, respectively, and the center of the concentric circle is located at the origin of the coordinates. Then, several measured points are employed to construct the real profile. Four feature points, which are evenly distributed on the outer and inner circles, must satisfy the outer–inner–outer–inner principle. The remaining measured points are randomly distributed between the outer circle and inner circle. In this simulation, fifty points have been built, as shown in Table 1. Then, all the coordinates are imported to the progressive search method. Through the computation of MATLAB program, the coordinate of the minimum zone circle center is (0, 0) and the roundness error is 10 μm. The control points and roundness error of MZC obtained by the proposed method and other commonly used methods are demonstrated in Table 2. The computation time and iterations of the proposed method are less than those of the other methods. The result demonstrated that the proposed method is effective and accurate.

### 5.2. Performance Verification by the Comparison Experiments

Two published datasets are applied to verify the correctness, effectiveness, and reliability of this method. The first dataset, which has regular 39-coordinate data [8,32], is used to determine the performance of the proposed method, as shown in Table 3. The roundness error of the reference [32] is 8.5 μm. The roundness error calculated by the progressive search method is determined to be 8.5 μm as well, as shown in Table 4. The results are completely consistent with that in the publication.

The second dataset was published in [20], which has 32 coordinate data. The roundness error of the reference is 7.218 μm. The roundness error of the proposed method is determined to be 7.218 μm (retain three decimal places), as shown in Table 5. Compared with the published results in the publication, the two results are completely consistent.

The proposed method and the methods of [20,32] are fully satisfied with the definition of the roundness error according to ISO standard (i.e., minimum zone criterion). Therefore, the roundness errors determined by the proposed method are the same as the results published in the references [20,32]. The computation time and iterations are not provided in [32]. By the proposed method, the computation time is 0.022 s and the iterations are five times. The reference [20] provides the computation time (0.109 s) but no iterations. By the proposed method, the computation time is 0.047 s and the iterations are seven times. The computational efficiency of the proposed method is better than one of the reference [20].

## 6. Analysis of the Algorithm Convergence and Optimal Solutions

According to the definition of the roundness error, it is well understood that the radius difference of concentric circles is gradually decreasing whilst the reference center of concentric circles moves from the LSC center to the MZC center. Suppose the roundness error of the reference center is regarded as an objective function, the function value is gradually decreasing during the approach process. The LSC center can be determined easily. A search circle model is established based on the LSC center to determine the search direction. The bisection method used to determine the radius of the search circle model to be *E*_0_/2, which is the half value of the minimum extreme difference of the last step, at every step when the feature points of the search circle excessively deviated from the MZC center, the search scope can be rapidly contracted.

By comparing the roundness error of the feature points on the search circle, it can avoid being deviated from the right direction. If the minimum function value of the feature points in each step is denoted as *f*(*x_i_*), where *i* is the number of iterations. Then, the search principle of the algorithm is as follows:

(4) $$f(x_{0})\geq f(x_{1})\geq f(x_{2})\geq ...\geq f(x_{n})\geq ...$$

where Equation (4) can satisfy the requirement of fast convergence as well. The analysis above is also mentioned in optimization theory, that is, by proving that the ratio of the preceding and following values increases or decreases monotonically, the search interval is continuously shortened to obtain an approximation of the minimum point [33]. Then, the convergence value is employed to obtain the MZC center, as shown in Section 4.4.

## 7. Discussion

The 2+2 model is satisfied with the MZC criterion, but it is not easy to directly determine four control points. Using the progressive search method can easily choose four control points from all the measured points with less time and fewer iterations, and then the MZC center and roundness error are determined. The aforementioned results indicate the progressive search method has good abilities in terms of accuracy, efficiency, and reliability. The programs developed by MATLAB were run on a personal computer sourced by Hewlett-Packard manufacturer in Palo Alto city, USA with a 2.60 GHz Intel Core i7-9750H processor and 8.00 Gigabytes of memory. The real-time monitoring on software shows that the computation time and the iterations of all the experiments are no more than 0.1 s and 10 times, respectively. As a result, the progressive search method can improve the computational efficiency.

Furthermore, as the search scope can be contracted rapidly, the progressive search method has a good ability for fast convergence in global searching, so the search strategy applied to the evaluation of sphericity, cylindricity, and other geometry form errors can be discussed further. Research jobs are ongoing.

## 8. Conclusions

To match the strict requirement during the process of roundness evaluation, this paper proposes a progressive search method for minimum zone circle evaluation. The core of the search method is to determine the four control points of the MZC mathematic model, i.e., 2+2 model. The evaluation process is easy to operate. Simulation and comparison experiments are performed to verify the accuracy, efficiency, reliability, and computational efficiency of the proposed method. The proposed method can accurately identify the MZC center with a good ability in terms of convergency and time efficiency. As the exact center is searched step by step, the proposed method exhibits a good performance for roundness error evaluation.

## Figures and Tables

**Table 1:** Coordinates of measured points.

No.	x	y	No.	x	y	No.	x	y
1	1.010000	0.000000	18	−0.064701	1.007773	35	−0.998420	−0.128732
2	1.000803	0.096546	19	−0.160742	0.994247	36	−0.976229	−0.222818
3	0.988295	0.192470	20	−0.255783	0.975410	37	−0.949260	−0.315176
4	0.967235	0.287070	21	−0.346861	0.942534	38	−0.919532	−0.407049
5	0.927425	0.375473	22	−0.435926	0.905209	39	−0.873940	−0.492194
6	0.889292	0.463943	23	−0.521299	0.859938	40	−0.828374	−0.577838
7	0.838475	0.545787	24	−0.598111	0.801414	41	−0.768599	−0.654318
8	0.783360	0.624709	25	−0.674110	0.742270	42	−0.702507	−0.725396
9	0.723523	0.700693	26	−0.745823	0.677337	43	−0.625277	−0.784072
10	0.652907	0.766942	27	−0.805452	0.601124	44	−0.549904	−0.844800
11	0.577139	0.827372	28	−0.860674	0.521746	45	−0.466683	−0.894544
12	0.493576	0.876394	29	−0.903742	0.435219	46	−0.377509	−0.932455
13	0.405069	0.915059	30	−0.939770	0.345844	47	−0.284528	−0.958668
14	0.318016	0.957813	31	−0.971895	0.254861	48	−0.192849	−0.990236
15	0.224302	0.982731	32	−0.990760	0.160178	49	−0.096929	−1.004773
16	0.128243	0.994626	33	−1.005810	0.064575	50	0.018471	−1.005492
17	0.032226	1.004920	34	−1.007285	−0.032302			

Unit: mm.

**Table 2:** Comparison results obtained by the proposed method and other common methods.

Method	Control Points on the Outer Circle	Control Points on the Inner Circle	Roundness Error	Coordinates of the Center of the Circle	Computation Time	Iterations
LSC	40	47	0.010100	(0.000254, 0.000027)	≈0.000 s	\
PSO	40	24	0.010000	(0, 0)	0.467 s	78
Proposed method	1, 40	24, 47	0.010000	(0, 0)	0.031 s	5

Unit: mm.

**Table 3:** Coordinates of sampling points from the reference [8,32].

Number	x	y	Number	x	y	Number	x	y
1	1.0249	0.0863	14	−0.9394	0.1561	27	−0.4635	−0.9195
2	0.9991	0.2226	15	−0.2071	0.9218	28	0.4736	−0.9507
3	0.5974	0.7736	16	−0.3381	0.8782	29	0.5942	−0.8781
4	0.4731	0.8485	17	−0.4643	0.8132	30	−0.2059	−1.0269
5	0.8803	0.4794	18	−0.5771	0.7369	31	0.9950	−0.3272
6	0.8017	0.5899	19	−0.7763	0.5367	32	1.0218	−0.1921
7	0.9527	0.3551	20	−0.6838	−0.7485	33	−0.0686	−1.0512
8	0.7047	0.6884	21	−0.5795	−0.8424	34	0.0710	−1.0568
9	0.2101	0.9295	22	−0.9618	0.0170	35	0.2087	−1.0377
10	0.0708	0.9483	23	−0.9454	−0.2605	36	0.3445	−1.0078
11	−0.0683	0.9382	24	−0.9077	−0.3956	37	0.7082	−0.7982
12	−0.8432	0.4157	25	−0.8443	−0.5203	38	0.8873	−0.5832
13	−0.9022	0.2890	26	−0.7764	−0.6394	39	0.9510	−0.4578

Unit: mm.

**Table 4:** Results obtained by the proposed method.

Method	Control Points on the Outer Circle	Control Points on the Inner Circle	Roundness Error	Coordinates of the Center of the Circle	Computation Time	Iterations
Proposed method	15, 34	12, 32	0.0085	(0.0356, −0.0529)	0.022 s	5

Unit: mm.

**Table 5:** Results obtained by the proposed method and the reference [20].

Method	Control Points on the Outer Circle	Control Points on the Inner Circle	Roundness Error	Coordinates of the Center of the Circle	Computation Time	Iterations
Proposed method	2, 20	10, 26	0.007218	(0.012735, −0.000627)	0.047 s	7
Reference [20]	2, 20	10, 26	0.007218	(0.012735, −0.000627)	0.109 s	\

Unit: mm.

## Data Availability

The original data presented in this study can be requested from the corresponding author. The data are not available to the public for privacy reasons.
